# Royal Medical and Chirurgical Society

**Published:** 1897-05-29

**Authors:** 


### Medical Progress and Hospital Clinics.

[*The Editor will be glad to receive offers of co-operation and contributions from members of the profession. All letters should be addressed to* The Editor, at the Office, 28 & 29, Southampton Street, Strand, London, W.C.]

### ROYAL MEDICAL AND CHIRURGICAL SOCIETY.

At the meeting last Tuesday, Mr. Clutton read a paper on a Case of Subclavian Aneurism successfully treated by ligature. The patient was a spare, healthy man aged 54. There was a small sacculated aneurism in the third part of the right subclavian artery, reaching from the outer border of the scalenus auticus to the clavicle. The heart and aorta were stated to be free from obvious signs of disease. On March 18th, 1896, an animal ligature was applied on the proximal side at the junction of the first and second part of the subclavian artery. The ligature was tied as a "stay-knot" with sufficient tightness to arrest the circulation, but without rupturing the coats. The superior intercostal was also tied at the same time and in the same manner. At the end of five or six weeks the aneurism and the radial artery were pulsating as they did before the operation. On June 10th the first part of the subclavian artery was exposed and tied with floss silk on the proximal side of the first ligature. The internal mammary and thyroid axis were also tied at the same time. The stay-knot was employed for each ligature, and the walls of the vessels were approximated without sufficient tightness to rupture the coats. Pulsation in the aneurism and in the radial artery ceased as the ligature on the main vessel was being tightened. On June 17th the aneurism was again pulsating. The first part of axillary was therefore immediately tied in the same manner with floss silk, so as to cut off all the collateral vessels from the intercostals in the axilla. After this operation the aneurism ceased to pulsate, and gradually disappeared. The patient left the hospital on August 8th without any swelling above the clavicle. It was thought probable that if the first ligature had been of silk instead of an animal material the subsequent operations might have been unnecessary.

